# Diagnosis of cardiac abnormalities based on phonocardiogram using a novel fuzzy matching feature extraction method

**DOI:** 10.1186/s12911-022-01976-6

**Published:** 2022-09-02

**Authors:** Wanrong Yang, Jiajie Xu, Junhong Xiang, Zhonghong Yan, Hengyu Zhou, Binbin Wen, Hai Kong, Rui Zhu, Wang Li

**Affiliations:** School of Pharmacy and Bioengineering, Chongqing University of Technology, Chongqing, China

**Keywords:** Feature engineering, Phonocardiogram, Machine learning, Wavelet, Matching feature extraction

## Abstract

**Background:**

The diagnosis of cardiac abnormalities based on heart sound signal is a research hotspot in recent years. The early diagnosis of cardiac abnormalities has a crucial significance for the treatment of heart diseases.

**Methods:**

For the sake of achieving more practical clinical applications of automatic recognition of cardiac abnormalities, here we proposed a novel fuzzy matching feature extraction method. First of all, a group of Gaussian wavelets are selected and then optimized based on a template signal. Convolutional features of test signal and the template signal are then computed. Matching degree and matching energy features between template signal and test signal in time domain and frequency domain are then extracted. To test performance of proposed feature extraction method, machine learning algorithms such as K-nearest neighbor, support vector machine, random forest and multilayer perceptron with grid search parameter optimization are constructed to recognize heart disease using the extracted features based on phonocardiogram signals.

**Results:**

As a result, we found that the best classification accuracy of random forest reaches 96.5% under tenfold cross validation using the features extracted by the proposed method. Further, Mel-Frequency Cepstral Coefficients of phonocardiogram signals combing with features extracted by our algorithm are evaluated. Accuracy, sensitivity and specificity of integrated features reaches 99.0%, 99.4% and 99.7% respectively when using support vector machine, which achieves the best performance among all reported algorithms based on the same dataset. On several common features, we used independent sample t-tests. The results revealed that there are significant differences (p < 0.05) between 5 categories.

**Conclusion:**

It can be concluded that our proposed fuzzy matching feature extraction method is a practical approach to extract powerful and interpretable features from one-dimensional signals for heart sound diagnostics and other pattern recognition task.

**Supplementary Information:**

The online version contains supplementary material available at 10.1186/s12911-022-01976-6.

## Background

The heart, as an exceedingly vital organ of humanity, pumps blood throughout our bodies with periodic systole and diastole, which is critical for the correct operation of physical functions. However, the frequency of cardiovascular disease has been steadily increasing in recent years [1]. It has been estimated that aberrant cardiovascular circumstances caused around 30% of the deaths of people with the disease worldwide [2, 3]. It has the potential to greatly increase the survival rate of heart disease patients by employing efficient and accurate diagnostic procedures. Early effective therapies utilizing some realistic diagnostic tools can also improve practitioners' abilities to prevent and heal cardiovascular disease [4]. The phonocardiogram (PCG) signal contains a wealth of early pathological information about cardiac valves and has been shown to be useful in the early detection of possible heart illness [5–7]. The creation of cardiac sounds is closely linked to the opening and closing of the atrioventricular, aortic, and pulmonary valves [8–12].

Traditional auscultations provide an assessment of cardiovascular problems based on the clinicians' expertise and knowledge [13]. However, this strategy is inefficient and prone to error [14]. With the advancement of computer technology, machine learning techniques, computer-aided techniques for the diagnosis of cardiovascular and other disorders, like COVID-19, are becoming increasingly frequent [15–19]. Deep learning is an important subfield of machine learning. The main advantage is that it can automatically extract features from the original signal and discover potential connections between data and prediction value [17]. It has also demonstrated good practicability and reliability in the field of speech recognition in recent years [20]. Simultaneously, a large range of deep learning models, including convolutional neural network (CNN), deep neural network (DNN), and recursive neural network (RNN), have demonstrated significant improvements in cardiovascular diseases diagnosis and recognition [16].

However, several drawbacks of deep learning are unavoidable. The first is the difficulty with data collecting and annotation [21]. To develop deep learning models, deep learning methods require a large number of labelled samples as training data. Nonetheless, the enormous workload generated by a large number of high-accuracy data capture and high-precision annotation is frequently undesirable to doctors and patients [21, 22]. The second difficulty is deep learning technology's reliance on strong processing capacity. The training time of the deep learning model increases dramatically as the amount of data increases. And when there is an inaccurate annotation in the annotation of data, it usually result in an extremely high error rate [22]. Furthermore, the poor interpretability of deep learning constituted a significant barrier [22]. In contrast, feature engineering may be a good solution to the problems that deep learning algorithms confront.

Feature engineering has long been an important strategy to using PCG to diagnose cardiac abnormalities. Weize Xu et al. produced a pediatric congenital heart sound database with 941 PCG signals for heart disorders. The researchers then devised a segment-based heart sound segmentation technique to mitigate the effects of local-nose. To classify data from 84 features, Random Forest and Adaboost classifiers were used. Their findings suggest that the best accuracy is 95.3% [13]. Mehmet Ali Kobat et al. used a new stable feature generation method to automatically diagnose cardiac valve problems. They extracted the 64 most discriminative features from neighbor-hood component analysis. KNN and SVM were introduced in the final classification, with 99.5 percent and 98.3 percent accuracy, respectively [23]. Pengpai Li et al. created a multi-modal feature based on PCG and ECG signals to diagnose cardiovascular diseases (CVDs). They used SVM as the classifier, and the AUC value of their model's highest performance is 0.936 [24]. Miguel et al. developed a method for separating PCG signals into silences and basic heart sounds. The segments were then joined with a simple genetic technique called differential evolution, and the results indicate a mean F1 score of 98.5% and 93.6% [25].

We concluded from researches described above that using heart sounds to identify cardiac disease is a hot topic in current research. However, several research employed complicated segmentation methods to separate the raw signals before classifying them using typical feature extraction strategies and machine learning classifiers. Despite the fact that they may obtain good diagnostic outcomes in their respective tasks, the complexity of segmentation algorithms and the inexplicability of deep learning have hampered their future clinical development. To solve this issue, we offer a unique feature extraction approach that does not need heart sound data segmentation. Furthermore, interpretable characteristics are employed to accurately and consistently diagnose cardiac disease.

In a previous study, we presented a discrete convolution wavelet transform (DCWT) for tracking accident signals in battery electric vehicles [26]. This paper proposes a fuzzy matching feature extraction method (FMFE) to extract matching features from heart sound signals by re-designing that algorithm. The following contents are divided into 4 sections: methodology, results, discussion, and conclusion. We provided the principles of the proposed method as well as the details of the experiment in the methodology section. The accuracy of the proposed approach and evaluation parameters were provided as results. In the discussion section, we also provided reasons for discussing the results. Finally, at the end of this work, conclusions are formed.

## Methodology

In our method, we first build a wavelet group that reflects the major correlation energy of a template signal. The fuzzy features of the target signals are then obtained by convolving them with an optimum collection of wavelets. Finally, fuzzy features are used to compute matching features between the template signal and the target signal. This work extracted self-matching features in the time domain, self-matching features in the frequency domain, and mutual matching features in the frequency domain.

**Fig. 1:**
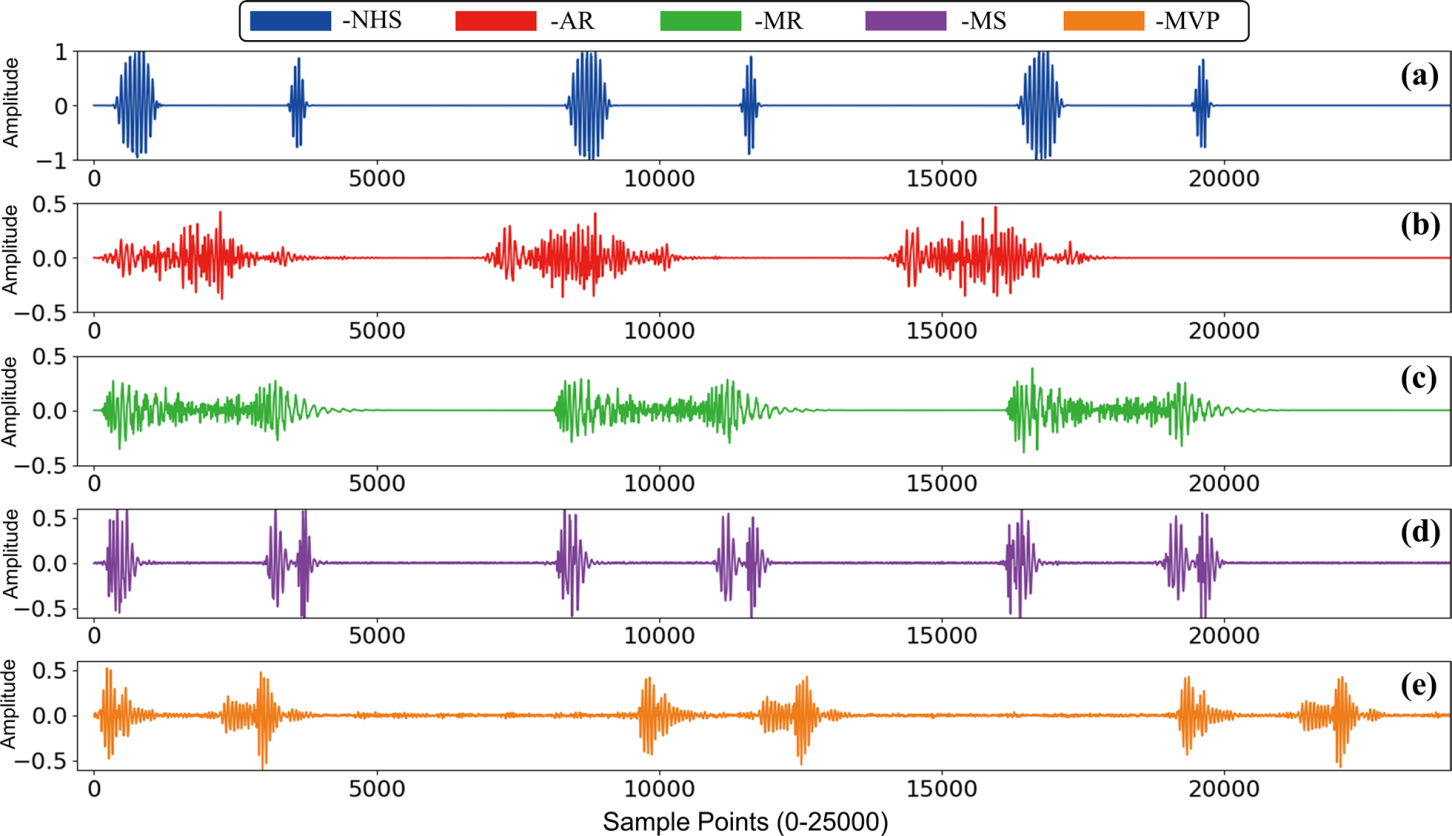
The PCG signals of five different cardiac diseases. **a**–**e** are the PCG of NHS, AR, MR, MS and MVP respectively. Each PCG signal contains 3 complete cardiac circles and sustain 2–3 s

The dataset we used in this study is from reference [8], which include five categories of PCG: normal heart sound (NHS), mitral stenosis (MS), aortic regurgitation (AR), mitral regurgitation (MR) and mitral valve prolapse (MVP). Typical representative of each category is shown in Fig. 1. They were gathered from a variety of sources, including books (Auscultation skills CD, Heart sound made easy) and websites (48 different websites provided the data including Washington, Texas, 3 M, and Michigan and so on). After excluding files with excessive noise, heart sound was sampled at 8000 Hz frequency rate and converted to mono channel, 3 period heart sound signal, data sampling, conversion, and editing were completed. The duration of each sample lasts 2 to 3 s containing 3 cardiac circles. Finally, there are 1000 samples in total, and each category holds 200 samples respectively. There are several benefits to using this dataset. The first comprises a large enough amount of data (1000 samples) and 200 samples for each analogy. Second, because it satisfies the sample balance property, it will not lead the algorithm to form preferences when the machine learning model is trained. Third, each item of data is labeled clearly. Fourth, this dataset has been used in several researches with positive experimental outcomes.

**Fig. 2:**
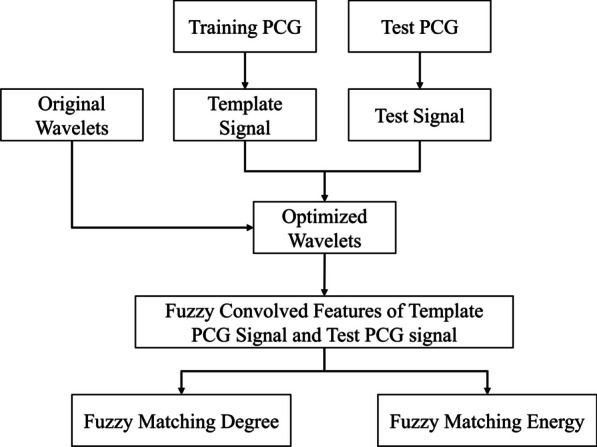
Flow chart of feature extraction in this study. Template PCG signal can be obtained from training PCG set. Then, Template PCG signal were utilized to optimize a group of initial Gaussian wavelets, and fuzzy convolved features of template PCG signal and test PCG signal can be computed. Further, we can have fuzzy matching degree and energy features

Procedures of FMFE are shown in Fig. 2. We got the template PCG signal from training PCG set and test signal from test PCG set. Then, a group of Gaussian wavelets were optimized from originally selected wavelets based on the template signal. Subsequently, template signal and test signal were convolved with these optimized wavelets, and fuzzy convolved features of template PCG signal and test PCG signal can be computed. Finally, based on these convolved features, fuzzy matching degree and fuzzy matching energy are obtained by matching computation.

### Acquisition of template feature

First of all, we need to clarify the following mathematic definition. For ease of more details in the proposed method and the ways of expression, the following formula representations will be used.

Given $x_{{\varvec{1}}}$ to $x_{{\varvec{n}}}$ are a set of vectors, so:

1 $$(x_{1} {\mathbf{;}}x_{2} {\mathbf{;}} \ldots {\mathbf{;}}x_{n} ) = \left( {\begin{array}{*{20}c} {x_{11} } & {{\varvec{x}}_{12} } & {\ldots } & {{\varvec{x}}_{1n} } \\ {{\varvec{x}}_{21} } & {{\varvec{x}}_{22} } & {\ldots } & {{\varvec{x}}_{2n} } \\ \vdots & \vdots & {} & \vdots \\ {{\varvec{x}}_{n1} } & {{\varvec{x}}_{n2} } & {\ldots } & {{\varvec{x}}_{nn} } \\ \end{array} } \right)$$

2 $$(x_{1} ,x_{2} ,\ldots ,x_{n} ) = \left( {\begin{array}{*{20}c} {x_{11} } & {{\varvec{x}}_{21} } & {\ldots } & {{\varvec{x}}_{n1} } \\ {{\varvec{x}}_{12} } & {{\varvec{x}}_{22} } & {\ldots } & {{\varvec{x}}_{n2} } \\ \vdots & \vdots & {} & \vdots \\ {{\varvec{x}}_{1n} } & {{\varvec{x}}_{2n} } & {\ldots } & {{\varvec{x}}_{nn} } \\ \end{array} } \right)$$

Given $x$ is a vector, then:

3 $$\left\| x \right\|^{2} = {\varvec{x}}_{1}^{2} + {\varvec{x}}_{2}^{2} + \cdots + {\varvec{x}}_{n}^{2}$$

4 $$Sqrt(x) = (\sqrt {{\varvec{x}}_{1} } ,\sqrt {{\varvec{x}}_{2} } ,\ldots ,\sqrt {{\varvec{x}}_{n} } )$$

As for $\odot$, given $x$ and $y$ are two vectors, then:

5 $$x \odot y = ({\varvec{x}}_{1} \cdot {\varvec{y}}_{1} ,{\varvec{x}}_{2} \cdot {\varvec{y}}_{2} ,\ldots ,{\varvec{x}}_{n} \cdot {\varvec{y}}_{n} )$$

Given $X$ and $Y$ are two matrixes, then:

6 $$X \odot Y = \left( {\begin{array}{*{20}c} {{\varvec{x}}_{11} \cdot {\varvec{y}}_{11} } & {{\varvec{x}}_{12} \cdot {\varvec{y}}_{12} } & {\ldots } & {{\varvec{x}}_{1n} \cdot {\varvec{y}}_{1n} } \\ {{\varvec{x}}_{21} \cdot {\varvec{y}}_{21} } & {{\varvec{x}}_{22} \cdot {\varvec{y}}_{22} } & {\ldots } & {{\varvec{x}}_{2n} \cdot {\varvec{y}}_{2n} } \\ \vdots & \vdots & {} & \vdots \\ {{\varvec{x}}_{n1} \cdot {\varvec{y}}_{n1} } & {{\varvec{x}}_{n2} \cdot {\varvec{y}}_{n2} } & {\ldots } & {{\varvec{x}}_{nn} \cdot {\varvec{y}}_{nn} } \\ \end{array} } \right)$$

Given $X$ is a matrix, *a* is a scalar, then:

7 $$X \odot \user2{a = }\left( {\begin{array}{*{20}c} {{\varvec{a}}x_{11} } & {{\varvec{a}}x_{12} } & \cdots & {{\varvec{a}}x_{1n} } \\ {{\varvec{a}}x_{21} } & {{\varvec{a}}x_{22} } & \cdots & {{\varvec{a}}x_{2n} } \\ \vdots & \vdots & \cdots & \vdots \\ {{\varvec{a}}x_{n1} } & {{\varvec{a}}x_{n2} } & \cdots & {{\varvec{a}}x_{nn} } \\ \end{array} } \right)$$

$FFT(x)$ represents the Fast Fourier transform for vector $x$.

As mentioned above, we used FMFE in 3 dimensions, which are self-matching in time domain, self-matching in frequency domain and mutual matching in frequency domain respectively. Thus, 3 matching templates are needed. In the time domain self-matching, the source of template is come from the PCG signal itself. Because the complete PCG signal are composed of 3 cycles in our dataset. In the dimension of self-matching in time domain, the template signal (***m***) is simply calculated by averaging all 3 cycles (***h***_***1***_***, h***_***2***_***, h***_***3***_) of one signal:

8 $${\varvec{m}} = \frac{{{\varvec{h}}_{1} + {\varvec{h}}_{2} + {\varvec{h}}_{3} }}{3}$$

In the dimension of self-matching in frequency domain and mutual matching in frequency domain, the template signal (***m***) is calculated by averaging Fast Fourier Transform (FFT) of all 3 cycles (***h***_***1***_, ***h***_***2***_, ***h***_***3***_) of one signal:

9 $$\user2{m = }\frac{{\left\| {FFT(h_{1} )} \right\|\user2{ + }\left\| {FFT(h_{2} )} \right\|\user2{ + }\left\| {FFT(h_{3} )} \right\|}}{3}$$

What is worth to be mentioned is that in mutual matching in frequency domain, ***h***_***1***_, ***h***_***2***_, ***h***_***3***_ are the 3 parts of one specific normal PCG (the shortest PCG in normal PCG dataset). In conclusion, all sample signals used the same template signal in mutual matching, but every sample signal has its own template signal in self-matching. After we have the template signal, we need to obtain the template features. ***W*** is an initial filter, it is a matrix constructed by N wavelets, which is described as ${(}{\varvec{w}}_{1} {;}{\varvec{w}}_{2} ;\ldots ;{\varvec{w}}_{N} )$. In this study, Gaussian wavelets were used. They are Gaussian 1th-8th high-derivative filters wavelets, N wavelets in total, and the length of each wavelet is L. N, and L are hyperparameters in FMFE. Then, the template features can be obtained by convolving the template signal with the initial filter matrix (***X*** is the template feature matrix, $\otimes$ is convolution operation):

10 $${\varvec{X}} = {\varvec{m}} \otimes \user2{W = }({\varvec{m}} \otimes {\varvec{w}}_{1} ;{\varvec{m}} \otimes {\varvec{w}}_{2} ;\ldots ;{\varvec{m}} \otimes {\varvec{w}}_{N} )$$

### Acquisition of correlation energy feature ***X***_***m***_ and ***X***_***s***_

However, not all the template features are usually needed to be considered. Because the same type of heart sound signals (for example, the same type of heart sound signals in normal people or the same type of heart sound signals in people with certain heart disease) come from different samples of individuals, and its features are not completely consistent. To reduce overfitting, an idea of fuzzy matching is proposed here. We consider that the same type of heart sound signal has the highest correlation energy. Therefore, a matching filter matrix based on the maximum correlation energy was constructed to extract the correlation energy features of the signals. We use a mask ***U*** to optimize initial filter ***W***:

11 $${\varvec{U}} = ({\varvec{\beta}}_{1} ,{\varvec{\beta}}_{2} ,\ldots ,{\varvec{\beta}}_{O} )$$

The ${\varvec{\beta}}_{1} ,{\varvec{\beta}}_{2} ,\ldots {\varvec{\beta}}_{O}$ are the eigenvectors corresponding to the top O eigenvalues of ***XX***^***T***^. Then, ${\varvec{W}}^{^{\prime}}$ can be optimized. They can be described as follows:

12 $${\varvec{W}}^{^{\prime}} = {\varvec{U}}^{T} {\varvec{W}} = ({\varvec{w}}_{{\varvec{1}}}^{\user2{^{\prime}}} ;{\varvec{w}}_{{\varvec{2}}}^{\user2{^{\prime}}} ;\ldots ;{\varvec{w}}_{O}^{\user2{^{\prime}}} )$$

where ${\varvec{w}}_{{\varvec{1}}}^{\user2{^{\prime}}} ;{\varvec{w}}_{{\varvec{2}}}^{\user2{^{\prime}}} ;\ldots ;{\varvec{w}}_{O}^{\user2{^{\prime}}}$ represent the optimized O filters (Because we only selected O eigenvectors to optimize the filter, the number of optimized filters becomes one of the hyperparameters O of the proposed method). Correspondingly, the fuzzy feature of template signal (***X***_***m***_) and of target signal (***X***_***s***_) can be obtained as follows:

13 $${\varvec{X}}_{m} = {\varvec{m}} \otimes {\varvec{W}}^{\user2{^{\prime}}} = ({\varvec{m}} \otimes {\varvec{w}}_{{\varvec{1}}}^{\user2{^{\prime}}} ;\,{\varvec{m}} \otimes {\varvec{w}}_{{\varvec{2}}}^{\user2{^{\prime}}} ;\,\ldots ;{\varvec{m}} \otimes {\varvec{w}}_{O}^{\user2{^{\prime}}} ) = ({\varvec{x}}_{{m{\varvec{1}}}} ;\,{\varvec{x}}_{{m{\varvec{2}}}} ;\,\ldots ;\,{\varvec{x}}_{mO} )$$

14 $${\varvec{X}}_{s} = {\varvec{s}} \otimes {\varvec{W}}^{\user2{^{\prime}}} = ({\varvec{s}} \otimes {\varvec{w}}_{{\varvec{1}}}^{\user2{^{\prime}}} ;{\varvec{s}} \otimes {\varvec{w}}_{{\varvec{2}}}^{\user2{^{\prime}}} ;\,\ldots ;\,{\varvec{s}} \otimes {\varvec{w}}_{O}^{\user2{^{\prime}}} ) = ({\varvec{x}}_{{s{\varvec{1}}}} ;\,{\varvec{x}}_{{s{\varvec{2}}}} ;\,\ldots ;\,{\varvec{x}}_{sO} )$$

### Acquisition of matching degree feature

Here we design a convolution to continuously compute matching degree between the template signal and target signal. To avoid the endpoint effect from convolution, the endpoints of ${\varvec{x}}_{mi}$ and ${\varvec{x}}_{si}$ were removed and then renamed as ${\varvec{x}}_{mi}^{\prime }$ and ${\varvec{x}}_{si}^{^{\prime}}$ respectively. Equations (15) and 16 described how we obtained the matching degree ***d***. ${\varvec{x}}_{si - norm}^{^{\prime}}$ is the norm of ${\varvec{x}}_{si}^{^{\prime}}$ in our convolutional computing, which is described in Eq. (17). Vector ***a*** in Eq. (17) are composed of the only element 1, which has the same size with ${\varvec{x}}_{mi}^{^{\prime}}$. $x_{mi - norm}^{^{\prime}}$ is the norm of template signal ${\varvec{x}}_{mi}^{^{\prime}}$, which is expressed in Eq. (18).

15 $${\varvec{y}}_{i} = \frac{{{\varvec{x}}_{si}^{^{\prime}} \otimes \overleftarrow {{{\varvec{x}}_{mi}^{^{\prime}} }} }}{{{\varvec{x}}_{si - norm}^{^{\prime}} \odot x^{^{\prime}}_{mi - norm} }},(i = 1,2,\ldots ,O)$$

16 $$\user2{d = y}_{1} \odot {\varvec{y}}_{2} ,\ldots , \odot {\varvec{y}}_{o}$$

17 $${\varvec{x}}_{si - norm}^{\prime } = Sqrt(({\varvec{x}}_{si}^{\prime } \odot {\varvec{x}}_{si}^{\prime } ) \otimes {\varvec{a}})$$

18 $$x_{mi - norm}^{^{\prime}} { = }\left\| {{\varvec{x}}_{mi}^{^{\prime}} } \right\|^{{2}}$$

### Acquisition of other matching features

We use max matching degree as one extracted feature in this study, we record the maximum value of matching degree ***d*** vector, which is recorded as $mmd_{{}}$. And record the point where the maximum value corresponds as the index of *mmd****.*** Based on index of the $mmd_{{}}$, corresponding energy features in ${\varvec{X}}_{s}$ can be easily found by taking the values according to the index position of ***mmd***. These values are referred as $e_{s1} ,e_{s2} ,\ldots ,e_{sO}$ in a vector ${\varvec{ME}}$:

19 $${\varvec{ME}} = (e_{s1} ,e_{s2} ,\ldots ,e_{sO} )$$

Each optimized wavelet gives a correlation energy value, so there are totally $O$ energy features in one cardiac cycle.

Because a template signal represents information of one cycle of each PCG signal, all 3 cycles of PCG in our study should have 3-folds of matching features. For each cycle, matching degree features and matching energy features are extracted according to the method above. This means that in one dimension of matching computation, each heart sound signal extracted $3 \times \left( {{\text{O}} + 1} \right)$ matching features. In the time domain, the self-matching degree features (TD.S.MD) and self-matching energy features (TD.S.ME) of one complete PCG signal can be expressed as follows:

20 $${\varvec{TD}}\user2{.S}\user2{.MD} = (mmd_{1}^{TD.S} , \,mmd_{2}^{TD.S} , \,mmd_{3}^{TD.S} )$$

21 $${\varvec{TD}}\user2{.S}\user2{.ME} = ({\varvec{ME}}_{1}^{TD.S} , \,{\varvec{ME}}_{2}^{TD.S} , \,{\varvec{ME}}_{3}^{TD.S} )$$

The extraction method of self-matching degree features and self-matching energy features in frequency domain is similar to the method above. The difference is that the heart sound signals in time domain of one heart beat cycle are transformed to the frequency domain using Fast Fourier Transform (FFT) and the template signal can be given in Eq. (9). Using the same method, we can have the following feature expression:

22 $${\varvec{FD}}\user2{.S}\user2{.MD} = (mmd_{{1}}^{FD.S} ,\,mmd_{2}^{FD.S} ,\,mmd_{3}^{FD.S} )$$

23 $${\varvec{FD}}\user2{.M}\user2{.MD} = (mmd_{1}^{FD.M} ,\,mmd_{2}^{FD.M} ,\,mmd_{3}^{FD.M} )$$

24 $${\varvec{FD}}\user2{.S}\user2{.ME} = ({\varvec{ME}}_{1}^{FD.S} ,\,{\varvec{ME}}_{2}^{FD.S} ,\,{\varvec{ME}}_{3}^{FD.S} )$$

25 $${\varvec{FD}}\user2{.M}\user2{.ME} = ({\varvec{ME}}_{1}^{FD.M} ,\,{\varvec{ME}}_{2}^{FD.M} ,\,{\varvec{ME}}_{3}^{FD.M} )$$

${\varvec{FD}}\user2{.S}\user2{.MD}$ is frequency domain self-matching degree feature of one complete heart sound signal, and ${\varvec{FD}}\user2{.S}\user2{.ME}$ is frequency domain self-matching energy features. ${\varvec{FD}}\user2{.M}\user2{.MD}$ and ${\varvec{FD}}\user2{.M}\user2{.ME}$ are mutual matching degree features and mutual matching energy features respectively.

### Classifiers

4 classifiers are used to evaluate obtained features in this study. Support vector machine (SVM) is a kind of generalized linear classifier. The decision boundary of SVM is the maximum margin hyperplane for learning samples. It utilizes hinge loss function to calculate empirical risk and adds regularization term to the solution system to optimize structural risk [27]. K-nearest neighbor (KNN) classification algorithm is one of the simplest methods in machine learning. The K nearest neighbors refers K nearest samples, which means that each category can be represented by its closest k neighbor’s category [28]. Random forest is a classifier in machine learning that contains multiple decision trees, which normally has well performance in machine learning task [29]. Multilayer Perceptron (MLP) is a classifier that follows the principle of human nervous system to learning and prediction. It uses the weight to store data, and uses the algorithm to adjust the weight and reduce the deviation in the training process [30]. Parameters of each classifier were optimized using grid search and the best ones are given in the Additional file1.

### Evaluation

In this paper, we used macro-recall (macro-R), macro-precision (macro-P) and accuracy to evaluate the performance of the method we proposed. Macro-R and macro-P are the assessment parameters often used in multi-classification. They are the average of the recall rate(R) and precision(P) obtained from each confusion matrix in our tenfold cross validation. These indicators are computed according to the following equation:

26 $$Recall = \frac{TP}{{TP + FN}}$$

27 $$P = \frac{TP}{{TP + FP}}$$

28 $$Sensitivity { = }\frac{TP}{{TP + FN}}$$

29 $$Specificity = \frac{TN}{{FP + TN}}$$

30 $${\text{macro - }}R = \frac{1}{n}\sum\limits_{i = 1}^{n} {R_{i} }$$

31 $${\text{macro - }}P = \frac{1}{n}\sum\limits_{i = 1}^{n} {P_{i} }$$

In equations above, TP, FP, FN and TN indicate true positive, false positive, false negative, true negative in confusion matrix respectively.

## Results

This paper proposed a unique FMFE approach for extracting matching features from heart sound signals. Various parameters can be selected (grid search) (N, L, O). The dimensions of signals after FMFE had been considerably decreased when compared to the original dimension of the heart sound signal (more than 16,000 dimensions of overall features).

**Fig. 3:**
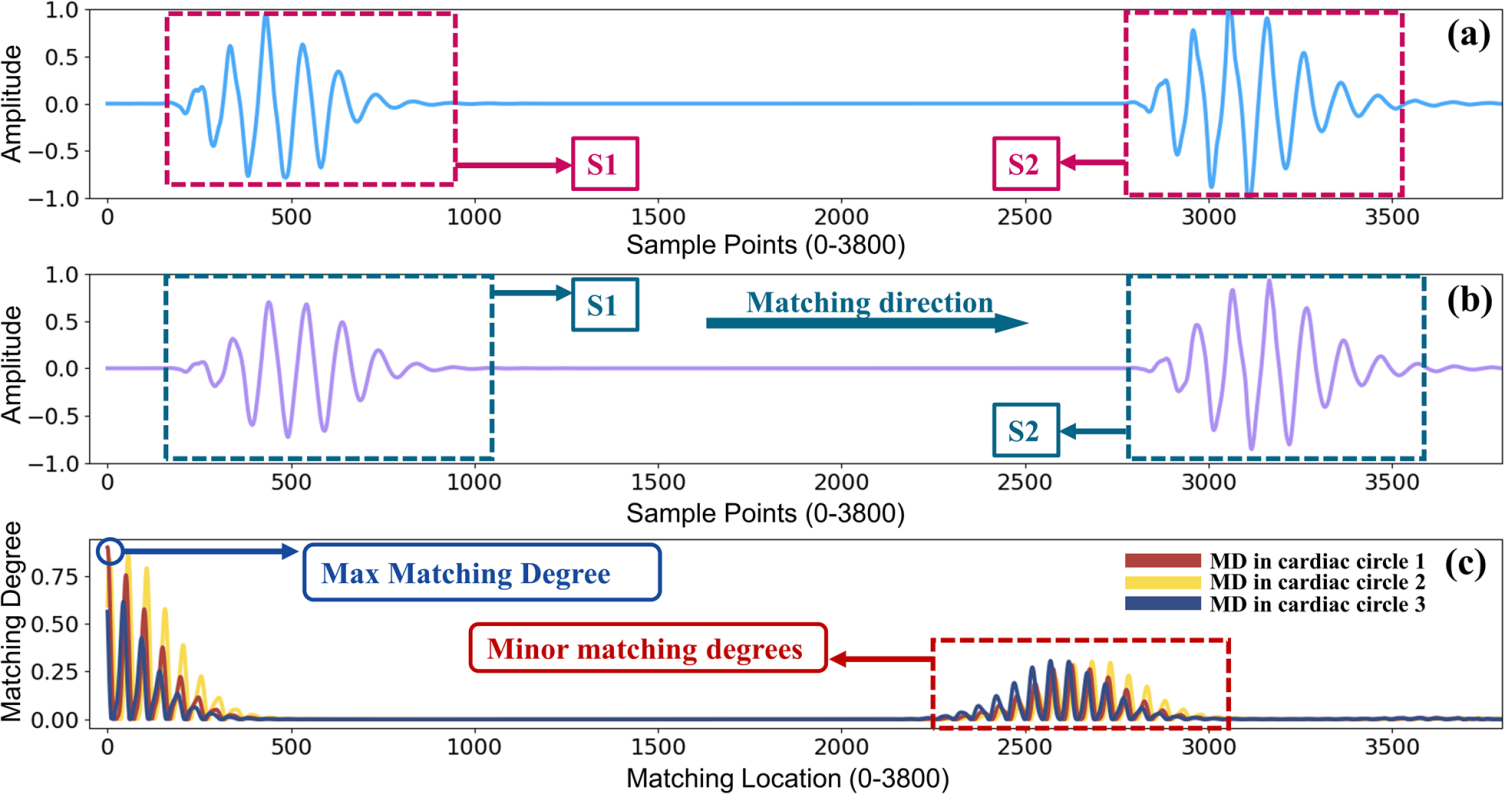
Matching results of TD.S.MD by FMFE. **a** is the example signal of one cardiac circle from NHS in time domain. **b** is the corresponding template signal and **c** is matching degree results obtained between original NHS signal (**a**) and the template signal (**b**). The maximum matching degree value can be obviously obtained at the initial position of matching process. As the template moves alongside matching direction, the matching degree gradually changes. However, the maximum value is still at the beginning matching position. Some minor degrees occurred due to the matching of S2 of original signal and S1 of template signal

Figure 3 shows the TD.S.MD feature results of a normal heart sound. Figure 3a gives one cardiac circle of a normal PCG signal in time domain from the data set. S1(the first audible part of heart sound signal) and S2 (the second audible part of heart sound signal) were marked clearly in this figure. Figure 3b shows the template PCG signal computed according to formula 1. Figure 3c illustrates the results of matching degree between the template signal and the original signal. As we can see in this figure, the max matching degree can be obtained at the very beginning of the matching, which means target signal matched tightly with template signal at the very beginning of the matching. When the template signal moves about 250 points, the matching degree dropped to near 0, which means two signals are no longer matched. When template signal moves to S2 of example signal, redundant matching degrees come out. The reason for the redundancy matching degree is that S1 of the template signal matched S2 of the example signal. As we can see, S1 of template signal and S2 of original signal have a certain similarity, so the second matching degree peak was obtained (Also called minor matching degree).

**Fig. 4:**
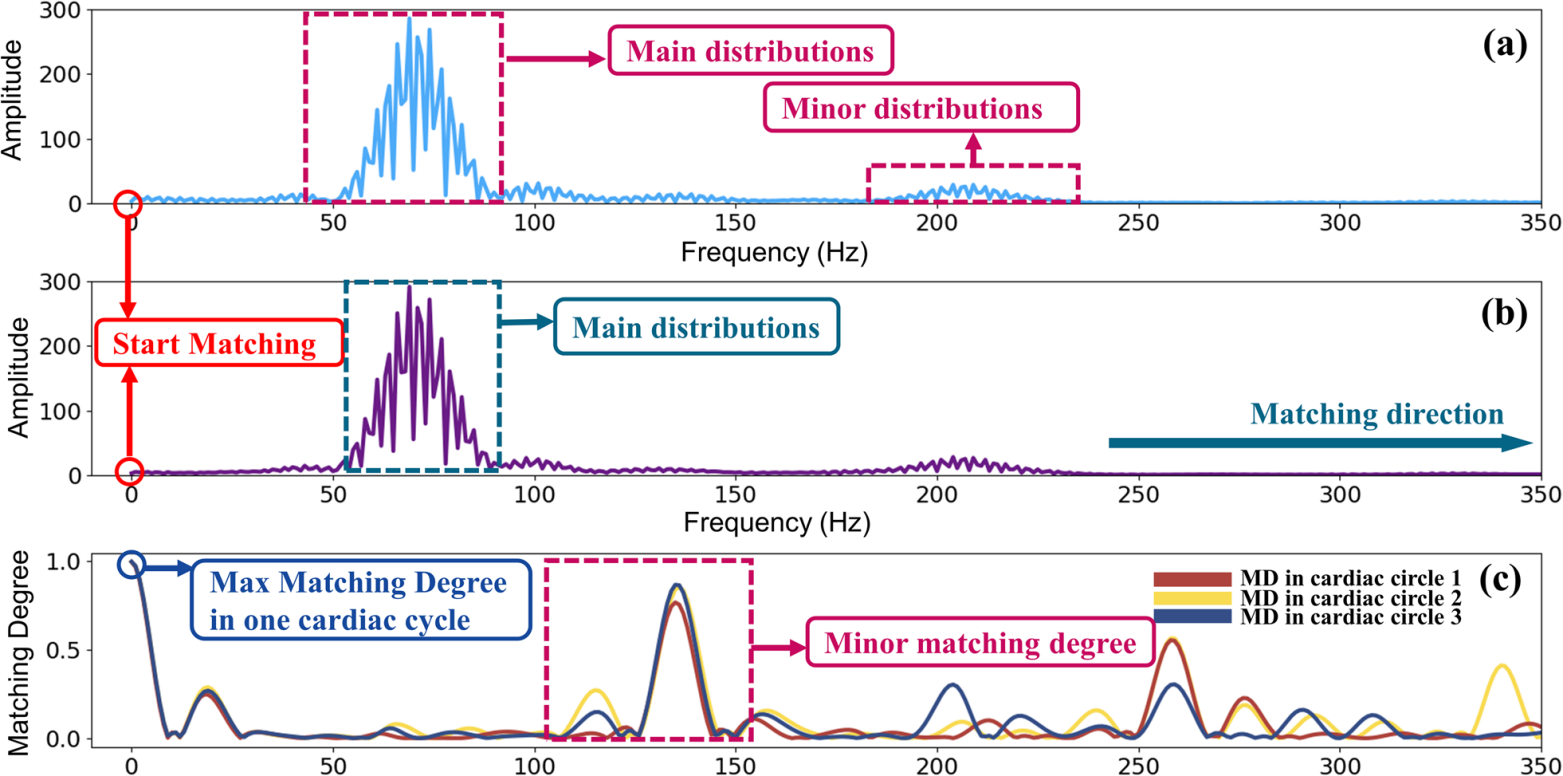
Matching results of FD.S.MD using FMFE. **a** is the modulus of PCG signals of one cardiac cycle in time domain after Fast Fourier Transform, **b** is the template signal in frequency domain and **c** is the matching degree results between **a** and **b**. The maximum FD.S.MD value can be easily obtained at the starting position of matching process. Some minor degrees appeared due to the matching of minor distributions of original signal and main distributions of template signal

Figure 4 illustrates the results of frequency domain self-matching degree (FD.S.MD). Figure 4a shows distribution of one cardiac cycle of an PCG signal after FFT process. Figure 4b shows the template signal computed based on Eq. (9). Figure 4c give the result of matching degree between these two signals. Similar to the result in time domain, the maximum matching degree is also obtained at the starting position followed by minor matching degrees. The reason can be explained similarly. Because one PCG signal has 3 cycles and every cycle should match with the template, thus curves of 3 matching degree are presented in Fig. 4c.

**Fig. 5:**
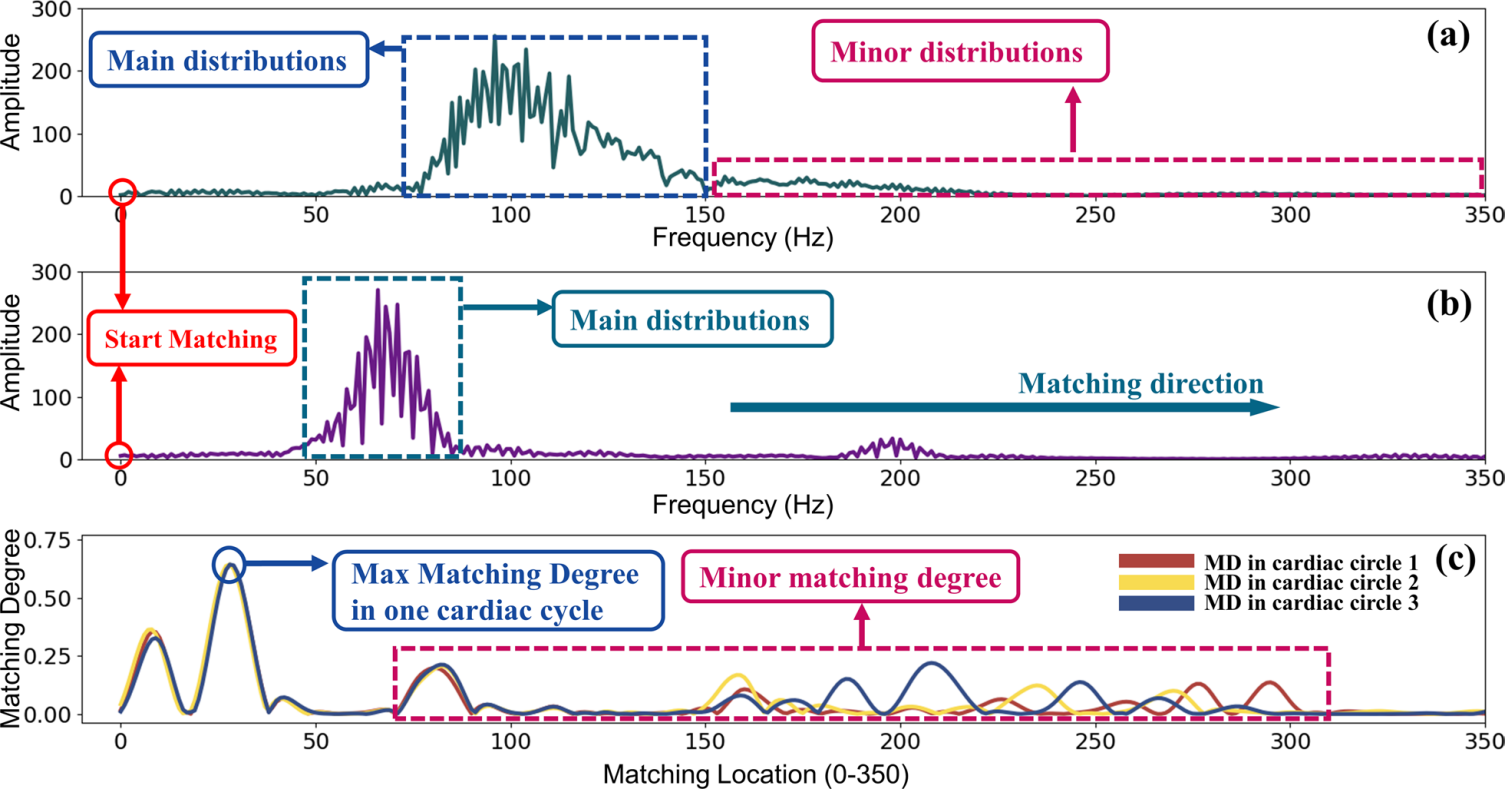
Matching results of FD.M.MD using FMFE. **a** is the modulus of heart sound of one cardiac cycle after FFT, **b** is the matching model signal in frequency domain and **c** shows the matching degree results

Figure 5 presents the results of FD.M.MD. There are some differences between self-matching and mutual matching in frequency domain. Figure 5a shows an example of abnormal PCG signal in the frequency domain. Figure 5b provides the template signal in frequency domain. It can be clearly found that main frequency components (main distributions) of these two signals are different. The max matching degree can no longer be obtained at the start position. Actually, it can be discovered at around 35 matching points. Similar to self-matching in frequency domain, 3 matching degree curves are also presented in Fig. 5c.

As we have described above, 4 classifiers are used to recognize 5 categories of samples based on features extracted using our proposed method. Classification results are shown in Table 1. Hyper-parameters of FMFE models are shown in Table 2. The best accuracy of 96.5% of independent FMFE features can be obtained by using random forest classifier.

**Table 1:** Classification results using different classifier

Features	Classifier	Macro-P (%)	Macro-R (%)	Accuracy (%)
	SVM	95.1	95.7	94.9
FMFE	KNN	90.3	90.5	90.0
	Random forest	96.7	97.0	96.5
	MLP	93.3	93.7	93.1

**Table 2:** Hyper-parameters for each type of features

Method	The type of features	N	L	O
	TD.S.MD	8	6	3
	TD.S.ME	8	6	3
FMFE	FD.S.MD	8	2	3
	FD.S.ME	8	2	3
	FD.M.MD	8	2	3
	FD.M.ME	8	2	3

## Discussion

We obtained the matching features by a matching operation between the template signal and the original signal. In the matching degree results described in Fig. 3c, at the beginning, the matching degree achieved the maximum value, and then gradually decreased to 0 in the fluctuation. The main reason is that the template signal of the normal PCG has no much difference (difference between their S1 is relatively small). So, the maximum matching degree can be gained at the very beginning of the matching. As the template signal moves along the matching direction, the S1 of the template signal and the S1 of the original signal gradually begin to stagger, so the matching degree will decrease rapidly during this process. When S1 of the template signal meets S2 of the original signal, due to the difference between the two and then minor matching degree are generated.

Similarly, the same principle in FD.S.MD of the matching degree distribution as shown in Fig. 4c. What’s worth to be mentioned here is that the interval between the maximum matching degree and the minor matching degree is not normally fixed. In Fig. 3c, the difference between the maximum matching degree and the minor matching degree is about 2000 points, but in Fig. 4c, the interval is only less than 100 points. Their previous distance is determined by the interval between the main distribution and the minor distribution in the original signal. Similar interpretations can be drawn for the characteristics of matching degree features in Fig. 5.

In this study, for this PCG dataset, FMFE showed the best results compared with other feature extraction methods. The method combined with the macro-precision, macro-recall and accuracy of the Random Forest classifier model reached 96.7% and 97.0% and 96.5% respectively. The recognition sensitivity and specificity reached 97.0% and 99.1% respectively. In 2018, Yaseen et al. [8] used the DWT algorithm to extract the features from the database used in this study, and their method combined the SVM model reaches an accuracy of 92.3%. The performance of the algorithm proposed in this study has surpassed DWT used by [8], showing great potential. In 2019, Ali Mohammad et al. [31] established a recognition model based on the same PCG database using PCA and random forest algorithms. Their accuracy reaches 94.8% which is also lower than the results of our study.

Wei Zeng et al. used the Teager–Kaiser energy operator (TKEO) and rational dilation wavelet transform (RDWT) methods to extract the instantaneous energy features of PCG signals. The average accuracy on five classifications could reaches 98.1%. However, the large amount of computation is also its inevitable disadvantage [32]. Hamza Cherif et al. confirmed the important role of discrete wavelets (DWT) in analysis of PCG signal [33]. All those researches have indicated that the importance of wavelets in the analysis of PCG. Oher research based on fuzzy features can also have reliable and effective applications, indicating fuzzy features also hold powerful potential in prediction tasks [34]. Therefore, to a certain extent, it shows the rationality of using Gaussian wavelet to extract fuzzy features in our proposed FMFE method. The study by Vibha Aggarwal et al. on the performance of DCT and DWT to PCG analysis also supports our views [35].

In fact, the FMFE only utilizes the time and frequency domain pattern matching information of the PCG signal, which can improve the feature engineering quality of the signal by integrating with other features. Here, we consider fusing Mel Frequency Cepstral Coefficients (MFCC) into our extracted matching features and examine its effect. MFCC simulates the human auditory system [36] with Mel filters that are sparse at high frequencies and dense at low frequencies. MFCCs are cepstral parameters extracted from the Mel-scale frequency domain. The Mel scale is nonlinear and its relationship with frequency can be approximated by the following formula [36]:

25 $$Mel(f) = 2585 \times \log (1 + \frac{f}{700})$$

After Mel filtering, the heart sound signal (mainly in low frequency) is well preserved, and finally restored to the time domain through DCT, which can be regarded as discrete signal envelope. The MFCC feature describes the slow changing process of the signal [36]. The model based on the fusion features of FMFE and MFCC greatly improves the recognition effect. As shown in Table 3, the macro-precision, macro-recall and accuracy of SVM model for recognizing five types of PCG signals based on the fusion features have all achieved or over 99.0%. Yaseen et al. [8] indicated that when MFCC is used independently, the accuracy on SVM and KNN are 91.6% and 80.2% respectively. However, features combined with MFCC by using SVM can reaches an accuracy of 97.9%. Alqudah et al.utilized PCA for extracting features and random forest for classification to achieve an accuracy of 94.8%. Their highest accuracy of 98.2% occurred by using Deep WaveNet with sensitivity of 97.0% and specificity of 92.5%. Tariq used CNN to reach an accuracy of 98.7% with a sensitivity of 98.7% and specificity of 99.6% [37]. In this research, the best accuracy of 99.0% of fuzzy features can be obtained by using SVM classifier. This result has exceeded the results of other algorithms based on the same dataset (such as DWT + MFCC, deep wave net and CNN), and achieves the best performance in diagnosing cardiac diseases, as shown in Table 4.

**Table 3:** Classification results using fusion features by different classifiers

Feature	Classifier	Macro-P (%)	Macro-R (%)	Accuracy (%)
	SVM	99.1	99.4	99.0
FMFE + MFCCs	KNN	98.5	98.5	98.4
	Random forest	98.3	98.5	98.2
	MLP	97.1	97.0	96.9

**Table 4:** Comparison with related works in 5 years using the same heart sound dataset

References	Feature extraction	Classifier	Accuracy (%)	Sensitivity (%)	Specificity (%)
[8]	DWT + MFCCs	SVM	97.9	98.2	99.4
[8]	DWT	SVM	92.3	92.3	98.4
[8]	MFCCs	SVM	91.6	87.3	96.6
[5]	–	Deep wavenet	98.2	97.0	92.5
[31]	PCA	Random forest	94.8	94.7	98.7
[31]	PCA	KNN	91.6	91.5	97.9
[37]	–	CNN	98.7	98.7	99.6
This study	FMFE	SVM	94.9	95.7	98.8
	FMFE	KNN	90.0	90.5	97.5
	FMFE	Random forest	96.5	97.0	99.1
	FMFE	MLP	93.1	93.7	98.3
	FMFE + MFCCs	SVM	99.0	99.4	99.7
	FMFE + MFCCs	KNN	98.4	98.5	99.6
	FMFE + MFCCs	Random forest	98.2	98.5	99.6
	FMFE + MFCCs	MLP	96.9	97.0	99.2

–Denotes that there is no feature extraction method was used

**Fig. 6:**
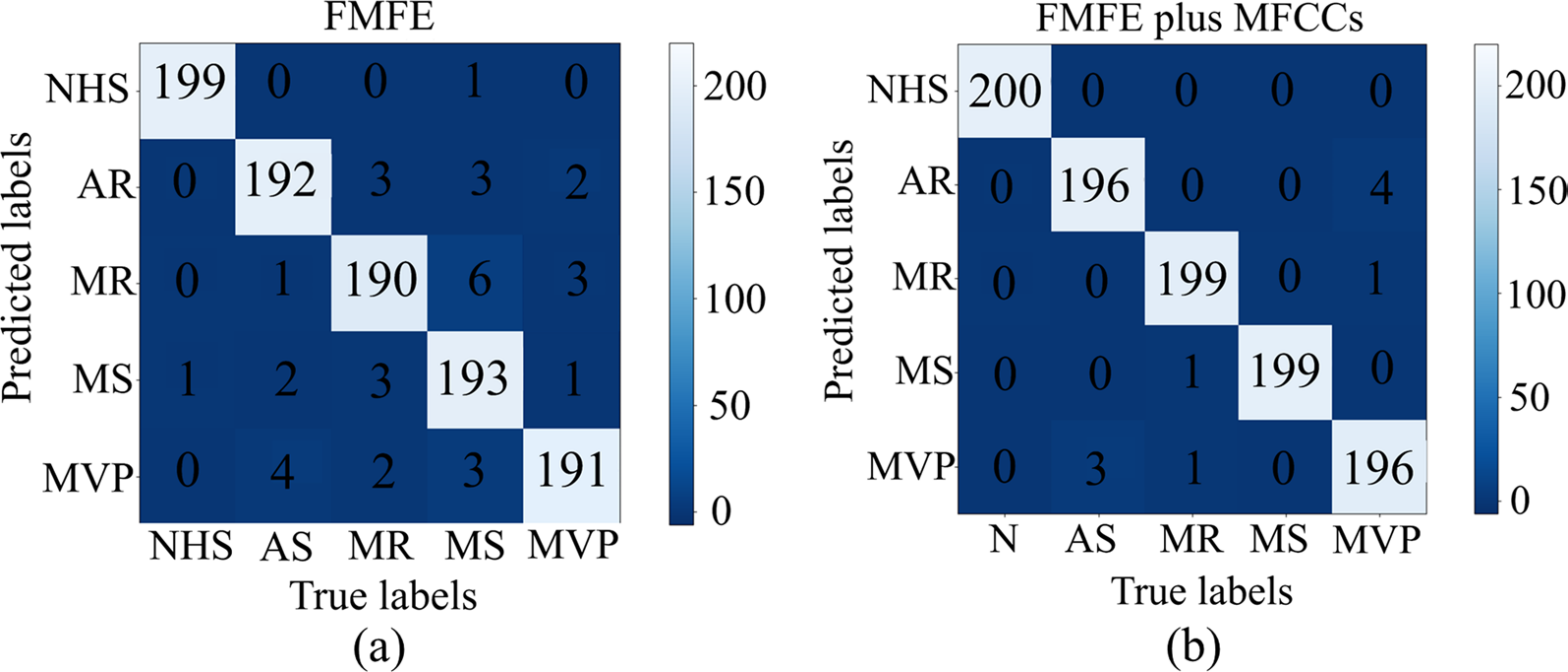
Best classifications confusion matrix of independent FMFE and FMFE plus MFCC. **a** illustrates that the best classification confusion matrix of independent FMFE from random forest. There is only 1 misclassification in NHS type in 200 test times. **b** shows that the best classification confusion matrix of FMFE plus MFCC from SVM. All the NHS samples are classified into right type. And other classification results hold a better performance than independent FMFE. **a** and **b** presented excellent results of this method

**Fig. 7:**
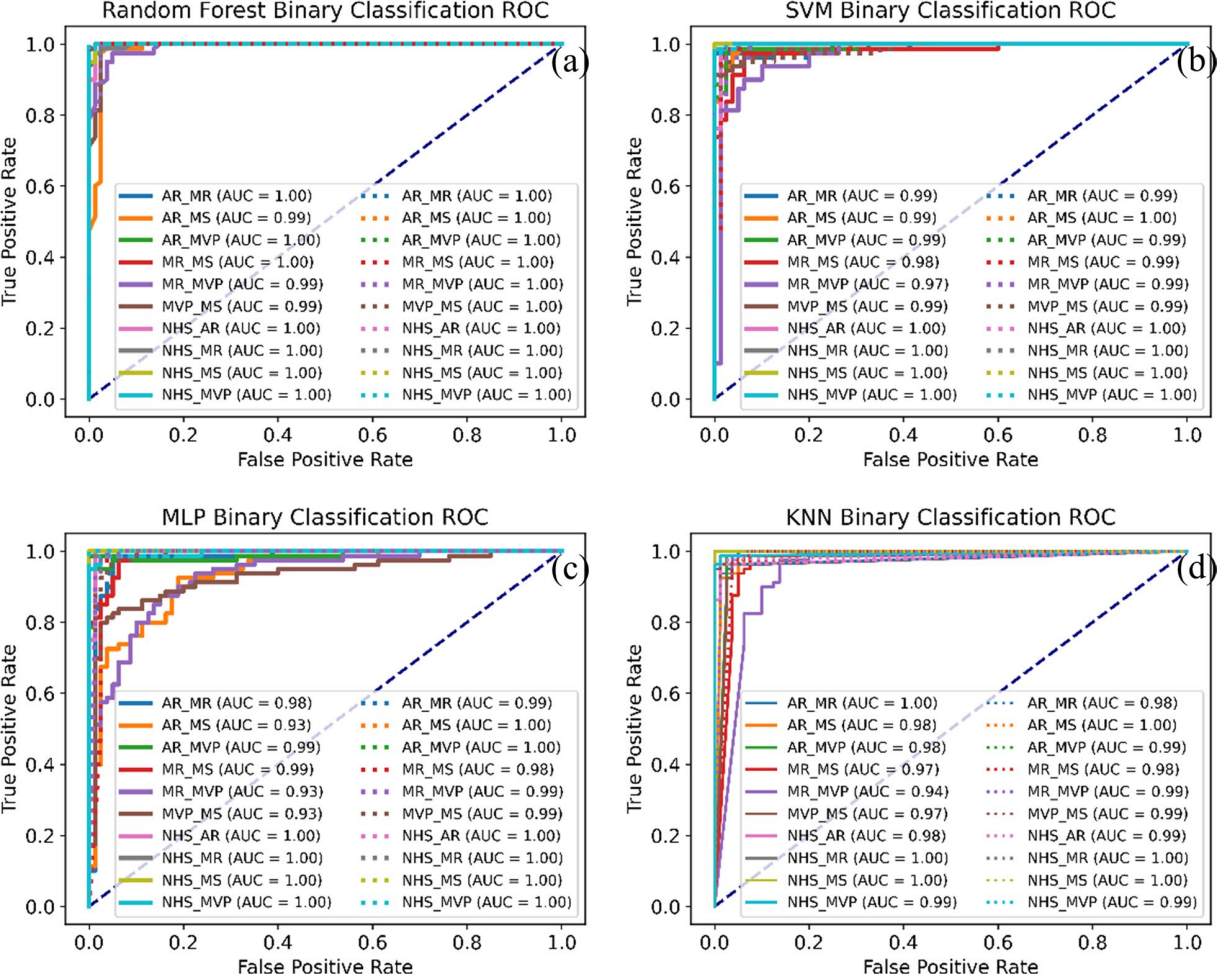
Binary classification receiver operating characteristic curve (ROC) of FMFE and FMFE plus MFCC. The solid line represents the binary ROC of the independent FMFE features, and the dashed line represents the binary ROC of the FMFE plus MFCC features. All the AUC value surpassed 0.97 showing advanced separability of the proposed features

Figure 6a shows that the best classification confusion matrix of independent FMFE from random forest. Only 1 misclassification in NHS type can be obviously found among 200 test times. And there are 10 misdiagnoses in MR type, which is the highest of 5 types. However, the right classification number of all five types is over 190 showing well performance. Figure 6b shows that the best classification confusion matrix of FMFE plus MFCC from SVM. All the NHS samples are classified into right type. And other classification results are also better than independent FMFE (Fig. 7).

**Fig. 8:**
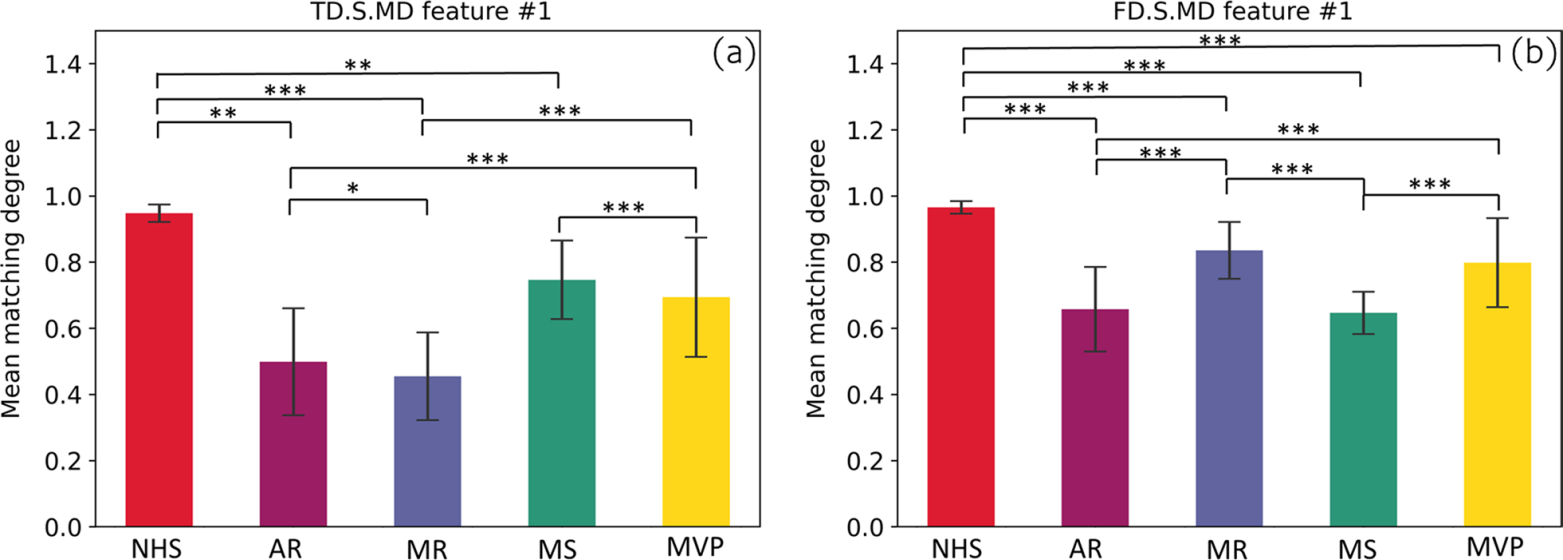
Statistical analysis of two typical features in FMFE. Independent sample t-test were used in this study. “***”, “**” and “*” referred p value less than 0.001,0.01 and 0.05 respectively. Standard error was employed to describe the dispersion of sample mean. Normal heart sound is always sustainable compared to cardiac abnormality types. There are significant differences between different types of the same features strongly support the classification results

In terms of the features itself we extracted by our method, the results of independent sample t-test showed that most of the extracted features usually had significant differences (p < 0.05). Take the statistical analysis results of the 2 features shown in Fig. 8 as an example. The significant difference between features is significant, and the significant difference p value between many categories is less than 0.001. The main reason is that different pathological features often have many unpredictable conditions. The stability of abnormal heart sound signal is poor, so the data will fluctuate greatly when matching and extracting features. On the contrary, the normal signal is often stable, so its fluctuation is relatively small. The significant difference between these features proves that there is good separability between different classes based on these features. At the same time, we use the extracted features to have binary classification among 5 categories. As shown by the ROC in Fig. 7, the classifiers all achieve excellent results (all AUC greater than 0.97). The same is true for the joint features of FMFE plus MFCC features. Therefore, the features extracted by our proposed FMFE method based on the heart sound signal are effective and reliable.

In general, we developed a one-dimension signal feature extraction algorithm based on fuzzy matching, and successfully improved the accuracy of abnormal cardiac diagnosis to a new level by integrating other features. It should be pointed out that different hyper parameter settings will lead to a different performance of the method. The improvement of parameter optimization efficiency is the future research direction we need to focus on.

## Conclusion

In this paper, a fuzzy matching feature extraction method for PCG signals is proposed. By combining with simple classifiers, features extracted by our proposed method show a potential performance in recognizing 5 categories of PCG signals. When integrated with MFCC features, the proposed feature extraction method obtained the best performance among all reported results based on the same dataset using feature engineering. With excellent interpretability and performance, our method may be promising in diagnosing cardiac diseases using machine learning techniques based on simple one-dimensional medical signals.

### Supplementary Information

**Additional file 1**. The selection of *β*. The document contains the selection process of β in the formula 4 in more details.

## Data Availability

The datasets analyzed during the current study are available in the yaseen21khan repository, https://github.com/yaseen21khan. And the main code of the proposed method can be access at https://gitee.com/yang1218/fmfe.

## Abbreviations

PCG: Phonocardiogram
CNN: Convolutional neural network
DNN: Deep neural network
RNN: Recursive neural network
KNN: K-nearest neighbor
SVM: Support vector machine
MLP: Multilayer perceptron
CVDs: Cardiovascular diseases
ECG: Electrocardiography
AUC: Area under curve
DCWT: Discrete convolution wavelet transform
FMFE: Fuzzy matching feature extraction
DCT: Discrete cosine transform
NHS: Normal heart sound
AR: Aortic regurgitation
MS: Mitral stenosis
MR: Mitral regurgitation
MVP: Mitral valve prolapse
FFT: Fast Fourier transform
TP: True positive
FP: False positive
FN: False negative
TN: True negative
TD.S.MD: Time domain self-matching degree
TD.S.ME: Time domain self-matching energy
FD.S.MD: Frequency domain self-matching degree
FD.S.ME: Frequency domain self-matching energy
FD.M.MD: Frequency domain mutual-matching degree
FD.M.ME: Frequency domain mutual-matching energy
ROC: Receiver operating characteristic curve
