# Correction: Diagnosis of cardiac abnormalities based on phonocardiogram using a novel fuzzy matching feature extraction method

**DOI:** 10.1186/s12911-023-02379-x

**Published:** 2023-12-08

**Authors:** Wanrong Yang, Jiajie Xu, Junhong Xiang, Zhonghong Yan, Hengyu Zhou, Binbin Wen, Hai Kong, Rui Zhu, Wang Li

**Affiliations:** School of Pharmacy and Bioengineering, Chongqing University of Technology, Chongqing, China

**Correction: Yang et al. BMC Medical Informatics and Decision Making (2022) 22:230**

10.1186/s12911-022-01976-6

After the publication of the original article [1], the authors reported an error in Eq. 1, which reads:

$$(x_{1} {\mathbf{;}}x_{2} {\mathbf{;}} \ldots {\mathbf{;}}x_{n} ) = \left( {\begin{array}{*{20}c} {x_{11} } & {{\varvec{x}}_{12} } & {\ldots } & {{\varvec{x}}_{1n} } \\ {{\varvec{x}}_{21} } & {{\varvec{x}}_{22} } & {\ldots } & {{\varvec{x}}_{2n} } \\ \vdots & \vdots & {} & \vdots \\ {{\varvec{x}}_{n1} } & {{\varvec{x}}_{n2} } & {\ldots } & {{\varvec{x}}_{n3} } \\ \end{array} } \right)$$

*Xn3* in the equation is incorrect as it should have been *Xnn*. Equation 1 should have been written as follows:

$$(x_{1} {\mathbf{;}}x_{2} {\mathbf{;}} \ldots {\mathbf{;}}x_{n} ) = \left( {\begin{array}{*{20}c} {x_{11} } & {{\varvec{x}}_{12} } & {\ldots } & {{\varvec{x}}_{1n} } \\ {{\varvec{x}}_{21} } & {{\varvec{x}}_{22} } & {\ldots } & {{\varvec{x}}_{2n} } \\ \vdots & \vdots & {} & \vdots \\ {{\varvec{x}}_{n1} } & {{\varvec{x}}_{n2} } & {\ldots } & {{\varvec{x}}_{nn} } \\ \end{array} } \right)$$

The original article [1] has been updated.
