# Supplementary material for: Diagnosis of cardiac abnormalities based on phonocardiogram using a novel fuzzy matching feature extraction method
Source: BMC Med Inform Decis Mak. 2022 Sep 2;22:230. doi: 10.1186/s12911-022-01976-6 (PMC9439280; doi:10.1186/s12911-022-01976-6)
Supplement: Supplementary file 1 — Additional file 1. The selection of β. The document contains the selection process of β in the formula 4 in more details. [file 12911_2022_1976_MOESM1_ESM.docx]

**Appendix**

**The selection of** $\beta$

Assume that the column vector $\beta=(b_{1};b_{2};...;b_{m})$ is the most relevant unit vector of $m\times n$ size matrix $X$ , then the relevant energy is defined as follows:

$$E=\frac{1}{2}\sum_{i=1}^{n} (\beta^{T}\cdot x_{i})^{2}=\frac{1}{2}\beta^{T}XX^{T}\beta$$

$x_{i}$ is the column vector of the matrix $X$ . Since $\beta$ is a unit vector, so:

$$\parallel\beta\parallel^{2}=1$$

Then the Lagrange equation under this constraint can be defined as:

$$L(\beta,\lambda)=\frac{1}{2}\beta^{T}XX^{T}\beta+\frac{1}{2}\lambda(1-\parallel\beta\parallel^{2})$$

Next, take the partial derivative of equation (3) with respect to $\beta$ :

$$\frac{\partial L(\beta,\lambda)}{\partial\beta}=XX^{T}\beta-\lambda\beta$$

Let its partial derivative be equal to 0, and equation (4) is deformed to obtain:

$$XX^{T}\beta=\lambda\beta$$

Equation (5) indicates that when the correlation energy is maximally obtained, $\beta$ must be the eigenvector corresponding to the maximum eigenvalue of the matrix $XX^{T}$.

By substituting Equation (5) into Equation (1), the maximum relevant energy can be obtained:

$$E=\frac{1}{2}\beta^{T}XX^{T}\beta=\frac{1}{2}\lambda\beta^{T}\beta=\frac{1}{2}\lambda$$

Above, the vector $\beta$ with the maximum energy related to $X$ is obtained. To maximize the correlation energy, $\beta$ must be the eigenvector corresponding to the maximum eigenvalue of the matrix$XX^{T}$.It can be observed from Equation (6) that the larger the eigenvalue is, the larger the corresponding correlation energy is, and the stronger the correlation between $\beta$ and $X$.
